# Dynamics of the Major Histocompatibility Complex Class I Processing and Presentation Pathway in the Course of Malaria Parasite Development in Human Hepatocytes: Implications for Vaccine Development

**DOI:** 10.1371/journal.pone.0075321

**Published:** 2013-09-25

**Authors:** Jinxia Ma, Stefanie Trop, Samantha Baer, Elian Rakhmanaliev, Zita Arany, Peter Dumoulin, Hao Zhang, Julia Romano, Isabelle Coppens, Victor Levitsky, Jelena Levitskaya

**Affiliations:** 1 W. Harry Feinstone Department of Molecular Microbiology and Immunology, Bloomberg School of Public Health, Johns Hopkins University, Baltimore, Maryland, United States of America; 2 Department of Oncology, School of Medicine, Johns Hopkins University, Baltimore, Maryland, United States of America; Centro de Pesquisa Rene Rachou/Fundação Oswaldo Cruz (Fiocruz-Minas), Brazil

## Abstract

Control of parasite replication exerted by MHC class I restricted CD8+ T-cells in the liver is critical for vaccination-induced protection against malaria. While many intracellular pathogens subvert the MHC class I presentation machinery, its functionality in the course of malaria replication in hepatocytes has not been characterized. Using experimental systems based on specific identification, isolation and analysis of human hepatocytes infected with *P. berghei* ANKA GFP or *P. falciparum* 3D7 GFP sporozoites we demonstrated that molecular components of the MHC class I pathway exhibit largely unaltered expression in malaria-infected hepatocytes until very late stages of parasite development. Furthermore, infected cells showed no obvious defects in their capacity to upregulate expression of different molecular components of the MHC class I machinery in response to pro-inflammatory lymphokines or trigger direct activation of allo-specific or peptide-specific human CD8+ T-cells. We further demonstrate that ectopic expression of circumsporozoite protein does not alter expression of critical genes of the MHC class I pathway and its response to pro-inflammatory cytokines. In addition, we identified supra-cellular structures, which arose at late stages of parasite replication, possessed the characteristic morphology of merosomes and exhibited nearly complete loss of surface MHC class I expression. These data have multiple implications for our understanding of natural T-cell immunity against malaria and may promote development of novel, efficient anti-malaria vaccines overcoming immune escape of the parasite in the liver.

## Introduction

Malaria remains a major global threat to human health and a leading cause of deaths worldwide (reviewed in 1). Significant ongoing efforts are focused on developing a protective vaccine capable of blocking transmission or preventing the onset of malaria infection (reviewed in 2,3,4,5). Successful completion of this task is unlikely to be achieved without detailed knowledge of host-parasite interactions at the cellular and molecular levels. However, very little is known about the effects of malaria parasite replication on the immuno- or antigenicity of infected host cells during the liver stage of infection.

*Plasmodium* sporozoites are transmitted through the bite of infected female *Anopheles* mosquitoes followed by sporozoite entry into the bloodstream and transit to the liver where they replicate and differentiate within hepatocytes (reviewed in 6,7). The liver stage of infection, which lasts 2 days in rodents and 6-8 days in humans, is asymptomatic and leads to subsequent release of merozoites from infected hepatocytes. The latter culminates in infection of red blood cells and clinical manifestations of malaria. Therefore, abrogation of the infection process at the asymptomatic liver stage is the most attractive goal of vaccination against malaria. Immunization with irradiated sporozoites can protect both experimental animals and humans against subsequent infection with live parasites (reviewed in 5,8,9,10) and this protective effect, at least in part, is accounted for by the activity of antigen-specific CD8+ T-cells [11,12,13,14,15,16,17,18], which prevent the development of parasites in the liver of the infected host. Although the phenomenon is well documented, the exact molecular mechanisms of CD8+ T-cell-mediated protection against malaria remain unclear ( [19,20,21] and reviewed in 22).

CD8+ T-lymphocytes recognize MHC class I: peptide complexes whose generation involves degradation of proteins by the proteasome, subsequent trimming of peptide fragments by intracellular proteases, peptide transport to the endoplasmic reticulum (ER) by the TAP1/TAP2 heterodimer and assembly of MHC class I heavy chains, β2m molecules and selected peptides into tripartite complexes. The latter step of the process is assisted by several chaperone molecules including tapasin, ERp57, calreticulin and calnexin followed by delivery of the complex to the cell surface (reviewed in [23,24,25,26,27]). Recognition of MHC class I complexes by differentiated cytotoxic T-lymphocytes (CTLs) triggers multiple effector functions characteristic of this cellular subset, including cytotoxic granule release [28,29,30,31] and expression of several death ligands [32,33,34,35,36,37,38], all capable of initiating programmed cell death in target cells, as well as secretion of a large panel of lymphokines and chemokines. Experiments in animal models revealed that many T-cell effector mechanisms, such as perforin release [20], expression of death receptor Fas [20], secretion of interferon gamma (IFNγ) [19] or tumor necrosis factor alpha (TNFα) [21], are either redundant for or make highly variable contribution to vaccination-induced protection against malaria depending on a particular parasite/host combination. This variability may be determined, at least in part, by differences in the capacity of various malaria parasite species and strains to affect the antigenic properties of infected cells. Experimental evidence addressing this aspect of malaria parasite biology at the liver stage of infection is limited and primarily based on direct imaging of hepatocyte/T-cell interactions which lacks any quantitative power and is vulnerable to highly subjective interpretations.

CTL recognition of cells invaded by viral or bacterial pathogens is often compromised by downregulation of MHC class I expression on the surface of infected cells that is achieved through a variety of molecular mechanisms ranging from unspecific shutoff of cellular gene transcription to specific post-translational targeting of individual components of the MHC class I machinery by specialized pathogen-encoded proteins (reviewed in 39,40,41,42,43,44,45,46,47,48,49,50,51,52). It is conceivable that changes in the levels of MHC class I expression could have a strong influence on the outcome of interactions between malaria-infected hepatocytes and parasite-specific CTLs. However, the effects of malaria parasite replication on MHC class I expression have not been systematically studied in mouse or human hepatocytes. During past decade human hepatocellular carcinoma cell line HepG2 was widely used by the malaria research community to study exoerythrocytic development of the rodent parasite *P. berghei* in vitro. Due to the lack of the relevant human cellular model permissive for human pathogens *P. vivax* and *P. falciparum*, this combination was commonly used to study changes in human host cells induced by the developing malaria parasite. In contrast to HepG2, the HC-04 cell line, derived from human liver, was reported to allow for exoerythrocytic development of *P. falciparum* [53]. Here we demonstrate that, besides being permissive for *P. falciparum*, HC-04 cells support full development of *P. berghei* that results in the release of the terminal exoerythrocytic forms, merosomes. The latter, in combination with the ability to selectively identify, retrieve and analyze human cells infected with GFP-transgenic rodent or human parasites, provided a novel system for direct monitoring and detailed analysis of the previously uncharacterized MHC class I antigen presentation pathway in infected cells, as well as comparing changes inflicted by rodent and human parasites. Furthermore, our model systems allow detection and separation of terminal exoerythrocytic developmental stages of malaria parasite distinguished by the membrane surface phenotype compatible with immune escape from T-cell mediated surveillance.

## Materials and Methods

### Hepatocyte cell cultures

The HC-04 cell line generated from primary human hepatocytes [53] was obtained from ATCC (Manassas, VA, USA) and propagated in IMDM containing 2.5% FCS and supplemented with 100 units/ml penicillin, 100 µg/ml streptomycin and 2 mM L-glutamine (GIBCO®, Life Technologies, Grand Island, NY) (“complete medium”). Freshly isolated primary human hepatocytes were obtained from Celsis IVT (Halethorpe, MD, USA) and were propagated in complete medium.

### Sporozoite Isolation

*Anopheles* *stephensi* and *Anopheles gambiae* mosquitoes were propagated and infected with malaria parasites in the Parasitology Core facility, the Johns Hopkins Malaria Research Institute. *P. falciparum* 3D7-GFP [54] parasite strain was generously provided by R. E. Sinden (Imperial College, London, UK). At day 21 (*P. berghei* ANKA GFP) or day 17 (*P. falciparum* 3D7-GFP) after exposure to infective blood meal, 300-800 female mosquitoes were anesthetized at -20°C, collected in 70% ethanol, washed in PBS and kept in complete medium on ice. The thorax, containing the salivary glands, was sequestered from each mosquito. All further procedures were carried out at +4°C. Homogenates of isolated material resuspended in complete medium were spun at 40 x *g* for 5 min and collected supernatants were further spun at 12,000 x *g* for 10 min (Sorvall RC-5C centrifuge, SS-34). The sedimented material was separated on an OptiPrep^TM^ Density Gradient (Sigma-Aldrich, St. Louis, MO) at 12,000 x *g* for 10 min (Sorvall RC-5C Plus, SS-34). Sporozoites collected from the gradient interface were washed once in complete medium and counted using a hemocytometer.

### Generation of “activated supernatant”

Epstein Barr virus (EBV) specific CD8+ cytotoxic T cells (CTLs) of different peptide epitope specificities [55,56] were exposed to an autologous lymphoblastoid cell line (LCLs) at a 10:1 ratio for 24 hrs at 37°C. The cultures were centrifuged at 10,000 x *g* and supernatant (1 ml per 1x10^6^ CTLs) containing soluble factors released by the activated T cells (“activated supernatant” or “AS”) was collected and stored in aliquots at -80°C. Control supernatants were collected following exposure of CTLs to HLA-mismatched LCLs that do not present the relevant EBV epitopes.

### Infection of human hepatocytes and cytokine treatment experiments

Hepatocytes were seeded onto collagen type I (BD Biosciences, San Jose, CA) coated 24-well plates and purified sporozoites were added at 1:1 sporozoite to hepatocyte ratio in complete medium. Plates were centrifuged at 380 x *g* for 5 min and, following incubation for 2 hrs at 37°C, extensively washed to remove uninvaded parasites. Cultures were further propagated under one of the following conditions: a) in complete medium alone or supplemented with recombinant IFNγ (1ng/ml) or TNFα (500 pg/ml); b) in the presence of 10% v/v of AS; c) in the presence of CD8+ CTLs preactivated on third party targets (1:100 CTL: hepatocyte ratio, generated and activated as described above. To block IFNγ and TNFα signaling, HC-04 cells were pretreated with 10 µg/ml of goat polyclonal IFNγ-receptor 1 neutralizing antibody (Sigma) and AS was preincubated with 10 µg/ml of IFNγ neutralizing mouse monoclonal antibody (Sigma) and 25 µg/ml of soluble TNF-R2:Fc chimera (Enbrel®). At 2 hrs after sporozoite infection, cells were washed to remove uninvaded parasites and anti-IFNγ-R1 antibodies were replenished at the same concentration.

### Isolation of infected cells

Separation of HC-04 cells infected with *P. berghei* ANKA GFP from uninfected cells present in the cultures exposed to sporozoites was done based on the expression of GFP in propidium iodide (PI)-negative alive hepatocytes at 24 and 48 hrs post-infection using a MoFlo Cell Sorter (Beckman Coulter, Indianapolis, IN). To verify the specificity of isolation, mRNA expression of parasite-specific genes was done as described below. To assess the purity of the GFP+ population, cells were stained with DAPI and typically 97-99% of isolated cells contained the characteristic pattern of parasite DNA staining in a parasitophorous vacuole as detected by fluorescence microscopy. The merosomes, identified at 46-48 hrs post-infection as a GFP^high^/SSC^low^ population and isolated using a MoFlo Cell Sorter, exhibited the characteristic pattern of multi-dotted DNA staining in the absence of host cell nuclei as detected by DAPI staining and fluorescence microscopy. Gating strategy for flow cytometry based cell sorting and side scatter characteristics of infected nucleated hepatocytes and merosomes is shown in each relevant figure.

### RT-PCR analysis of *P. berghei* liver stage parasites

mRNA expression of 18S, CSP and HSP70 *P. berghei* ANKA GFP genes was analyzed by RT-PCR in uninfected HC-04 cells along with GFP+ and GFP- cells isolated from infected cultures by flow cytometry based cell sorting at 24 hrs and 48 hrs post-infection. Reverse transcriptase reactions were performed with total cell lysates using the TaqMan® cells-to-CT kit (Applied Biosystems, CA, USA) and the resulting cDNA was then used as a template in PCR reactions with the following primer pairs:

P. berghei-CSP: 5’-aataataatgacgattcttata-3’/5’-caatattaaatatacttgaacat-3’;
P. berghei-HSP70: 5’-gtttacagaatcttctgtacaa-3’/5’-gttcatttataattctcataacat-3’;
P.berghei-18S: 5’-tgggagattggttttgacgtttatgt-3’/5’-aagcattaaataaagcgaatacatccttac-3’;
Human GAPDH: 5'-gcaaattccatggcaccgt-3'/5'-tcgccccacttgattttgg-3'

### Real-time PCR

Reverse transcriptase reactions were performed with total cell lysates of sorted cell populations using the TaqMan® Cells-to-CT kit (Applied Biosystems, CA, USA) followed by real-time PCR using commercially available TaqMan® gene expression assays (Table S1) and TaqMan® Master Mix (Applied Biosystems). All samples were analyzed in triplicates using a real-time PCR Applied Biosystems 7300 instrument. The thermal cycling profile for q-PCR reactions was the following: 2 min at 50°C, 10 min at 95°C, 40 cycles of 15 s at 95°C, and 1 min at 60°C. “No template” controls were included for all samples. The gene transcript levels were determined by relative quantitative PCR (qPCR) using comparative *C*T values and normalized using the average values of glyceraldehyde-3-phosphate dehydrogenase-specific signals in the same samples. Data are shown as expression units relative to GAPDH expression levels arbitrarily taken as 1 and represent the mean ±SD of triplicate reactions obtained from 4 to 9 individual infection and cell sorting procedures.

### Generation of DNA plasmids

The pVITRO2-neo-mcs (InvivoGen, CA, USA) contains two separate multiple cloning sites and allows the ubiquitous and constitutive co-expression of two genes of interest. Briefly, pBS vector containing mCD8-GFP fragment (Addgene, MA, USA, plasmid provided by Dr. Ligun Luo [57]) was digested with *XhoI/XbaI* and the mCD8-GFP fragment was inserted into pVITRO2-neo-mcs plasmid MCS1 site digested with *SalI*/*XbaI*. Sequence encoding mouse CD8α was PCR amplified from this vector with the following primers: forward, 5′-atgtatcgattgtcgagcaaaatg-3′; reverse, 5′-attacctaggtccagtgaaaagttcttc-3′, digested with *ClaI*/*AvrII* and inserted into pVITRO2-neo-mcs plasmid MCS1 site digested with *ClaI*/*AvrII*. Surface expression of mouse CD8α serves as a heterologous surface marker of transfection efficiency in human cells. Full length CSP from the *P. falciparum* 3D7 strain was first PCR amplified with the following primers: forward, 5’-aaaccacgtatattataaattacaa-3’; reverse, 5’-aactaagatgtgttctttatcta-3’ and inserted into pJET1.2/blunt Cloning Vector (Fermentas, MD, USA). Then, full length CSP gene from the *P. falciparum* 3D7 strain was PCR amplified from this vector with the following primers: forward, 5′-tataggatccggcgtaatacgactcact-3′; reverse, 5′-attagctagcctgatgaggtggttagca-3, digested with *BamHI*/*NheI* and inserted into pVITRO2-neo-mcs plasmid (containing mCD8α) MCS2 site digested with *BglII*/*NheI*. *P. falciparum* 3D7 “short” CSP was PCR amplified with the following primers from the same vector: forward, 5′-tataggatccatggcggatggtaatcctga-3′; reverse, 5′-tcagtctagatcctgatgaggtggttagca-3, digested with *BamHI*/*XbaI* and inserted into pVITRO2-neo-mcs plasmid (containing mCD8α) MCS2 site digested with *BglII*/*NheI*. This plasmid encodes the *P. falciparum* “short CSP” protein corresponding to the mature CSP form produced by proteolytic cleavage of full length CSP during parasite interaction with the host cell plasma membrane (Figure S1).

### MHC class I staining

HC-04 cells exposed to *P. berghei* ANKA GFP or *P. falciparum* 3D7 GFP sporozoites were incubated with HLA A,B,C-specific antibody (clone G46-2.6) or relevant IgG1 isotype control antibody (BD Pharmingen, San Diego, CA), both directly conjugated to allophycocyanin (APC). Expression of surface MHC class complexes on infected (GFP+) and uninfected (GFP-) hepatocytes was assessed by flow cytometry at the indicated time point post-infection for each experiment. Exclusion of dead cells was done using propidium iodide (PI). Data acquisition was performed using FACSCalibur and CellQuest acquisition software (Becton Dickinson, New Jersey, USA). Data were analyzed using FlowJo software (TriStar Inc., Ashland, Oregon, USA).

### Re-appearance of MHC class I complexes at the cell surface

HC-04 cells infected with *P. berghei* ANKA GFP for 24 hrs were incubated in a buffer containing 0.06 M sodium dihydrocarbonate and 0.113 M citric acid (pH 3.0) for 1 minute, followed by the quick adjustment of pH to 7.4 and extensive washing in complete medium. An aliquot was collected and placed on ice (“time zero” sample) whereas remaining cells were incubated in complete medium at 37°C, and aliquots were collected at 3, 6, 9, 12 and 15 hrs post treatment. Surface expression of assembled MHC class I complexes was simultaneously assessed in GFP+ and GFP- populations from the same infected cultures as described above.

### Transient transfection with plasmids encoding *P. falciparum* CSP

HC-04 cells were transfected with pVITRO2-CD8, pVITRO2-CD8/CSPfl, pVITRO2-CD8/CSPsh plasmids using Lipofectamine LTX (Life Technologies, Grand Island, NY). Expression of CSP was detected either by western blot in transfected cells at 24 hrs post transfection or by immunofluorescence using CSP-specific mouse monoclonal antibody 2A10 (kind gift of Dr. F. Zavala, JHMRI, JHU). Beta actin-specific antibody AC15 (Sigma-Aldrich, St. Louis, MO) was used to verify equal loading of cell lysates. Blots were developed using anti-mouse IgG-HRP (GE Healthcare, Pittsburgh, PA) and Super Signal West Dura Extended Duration Substrate (Thermo Scientific, Waltham, MA).

Twenty four and 48 hrs post transfection, surface expression of mouse CD8α was assessed by staining with anti-mouse CD8α-RPE or IgG2b-RPE isotype control antibody (Invitrogen, Grand Island, NY) and MHC class I expression was assessed in CD8α+ cell populations as described above. Data acquisition was performed using FACSCalibur (BD Biosciences, San Jose, CA). To monitor MHC class I pathway and its responsiveness to pro-inflammatory cytokines in CSP transfected cells, hepatocytes were sorted by MoFlo Cell Sorter (Beckman Coulter, Indianapolis, IN) 24 hrs post transfection, treated with AS or left untreated (as described above) for additional 24 hrs and real-time PCR analysis was performed for HLA A heavy chain, β2m and TAP1 genes at 48 hrs post transfection. In parallel, class I heavy chain expression was assessed by western blot using specific rabbit polyclonal antibody (kind gift of Dr. H. Ploegh).

To assess the subcellular pattern of CSP expression, HC-04 cells seeded on 8-well Lab-Tek^TM^ chamber slides (Nunc, Rochester, NY) were transfected with the control vector or CSPf.l plasmid. Twenty-four hours post-transfection, cells were fixed with 4% formaldehyde/0.02% glutaraldehyde for 15 minutes, followed by permeabilization in methanol for 10 minutes at 20°C, blocking 30 minutes in 1% goat serum in PBS, staining with anti-CSP antibody (1µg/ml), and anti-mouse IgG Alexa Fluor 488 (Molecular Probes, Life Technologies, Grand Island, NY). Slides were mounted with the SlowFade Gold AntiFade reagent with DAPI (Molecular Probes) and visualized on a Nikon 90i fluorescent microscope. Images were captured with a Hammamatsu-Orca camera attachment and analyzed using the Volocity software (PerkinElmer, Waltham, MA).

### T cell activation

A CD8+ HLA-A2-restricted cytotoxic T lymphocyte (CTL) clone specific for an EBV-derived CTL epitope GLCTLVAML (GLC) was used to assess T-cell activation induced by HLA-A2-positive HC-04 cells pre-pulsed with the synthetic GLC peptide. GFP+ and GFP- HC-04 cell populations were isolated by flow cytometry-based cell sorting at 6 hrs post-infection with *P. berghei* ANKA GFP parasites and propagated in complete medium for 18 hrs and T cell activation was evaluated at 24 hrs post-infection. Briefly, HC-04 cells pre-incubated with GLC peptide at concentration of 10^-6^ M for 1hr were co-cultured with CTLs at 3:1 T cell-to-hepatocyte ratio for 4h in medium containing Golgi Plug (BD Biosciences, San Jose, CA) without IL-2 followed by the concomitant surface staining with CD8-specific mouse monoclonal antibody conjugated to Pacific Blue and one of the following mouse monoclonal antibodies: anti-CD25, anti-CD69 or anti-41BB, all conjugated to phycoerythrin (PE). Cells were washed, fixed and permeabilized using Cytofix/Cytoperm (BD Biosciences, San Jose, CA) and incubated with one of the following antibodies: anti-IL-2, anti-IFNγ or anti-TNFα, all conjugated to allophycocyanin (APC). All antibodies were obtained from BD Biosciences (San Jose, CA). Samples were acquired with a LSR II flow cytometer (BD Biosciences) and analyzed using FlowJo software (Tree Star, Inc., Ashland, OR).

### Statistical analysis

All statistical analyses we done using paired Student’s *t* test. Differences were considered statistically significant if the *P*-value was ≤0.01 (see relevant figure legends).

## Results

### A novel system for monitoring the MHC class I antigen presentation pathway in human malaria-infected hepatocytes

**Figure 1:**
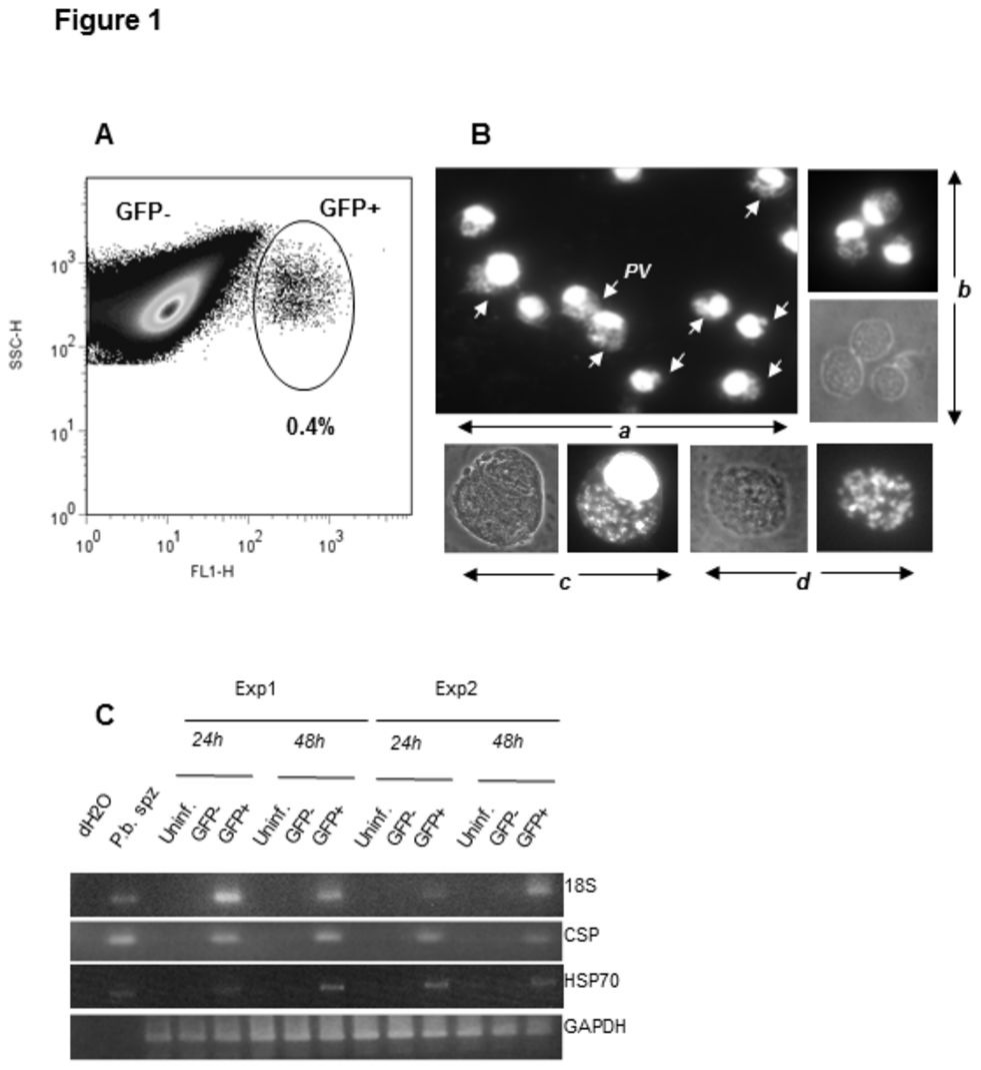
Flow cytometry-based detection of HC-04 cells infected with *P. berghei* ANKA GFP. (A) HC-04 cells infected with *P. berghei* ANKA GFP sporozoites were detected by flow cytometry based on GFP expression. (B) GFP+ cells were isolated at 24 hrs post-infection by flow cytometry-based cell sorting and stained with DAPI (a, b). Parasitophorous vacuoles (PVs) are indicated by arrows. (c) GFP+ cells isolated at 48 hrs post-infection and stained with DAPI contain large PVs and some cells (d) resemble merosome-like structures. (C) mRNA expression of *P. berghei* ANKA GFP genes *18S, CSP* and *HSP70* was analyzed by RT-PCR in GFP+ and GFP- cells purified by sorting from infected cultures. Samples were collected at 24 and 48 hrs post-infection. HC-04 cells from non-infected cultures were used as a negative control. Data from two independent experiments are shown in the figure.

A recent report has demonstrated that the human hepatocytic cell line HC-04 is susceptible to infection with *P. vivax* and *P. falciparum* sporozoites and supports their development into infectious merozoites within the time frame comparable to that observed *in vivo* during the liver stage of malaria infection. Although derived from normal hepatocytes, HC-04 cells exhibit apparently unlimited proliferative capacity, while possessing the cell surface phenotype and biochemical characteristics of normal liver parenchyma [53]. Thus, HC-04 cells represent a convenient alternative to primary hepatocytes as a model for *in vitro* analysis of parasite/host cell interactions during the liver stage of infection. However, the frequency of HC-04 cells productively infected with *P. falciparum* has been reported to reach a maximum of 0.06% ( [53] and our own observations). Moreover, detection of infected cells is further complicated by the proliferation of non-infected cells during the 6 to 8 day period required for *P. falciparum* to complete its sporozoite to merozoite transition. In search of a more convenient experimental system, we found, that besides *P. falciparum* and *P. vivax*, HC-04 cells are capable of supporting the development of exoerythrocytic forms of *P. berghei* whose natural reservoir is restricted to rodents. Recombinant *P. berghei* ANKA GFP sporozoites infected HC-04 cells with relatively high efficiency (0.2-0.4%, Figure 1A) as assessed by detection of GFP-expressing cells at 24-48 hrs post-infection. FACS-based separation of GFP+ and GFP- cells from the same cultures exposed to *P. berghei* ANKA GFP sporozoites showed that more than 95% of GFP+ cells contained a large parasitophorous vacuole (PV) at 48 hrs post-infection (Figure 1B, PVs are marked by arrows), while the GFP- population did not contain infected cells as determined by fluorescence microscopy or RT-PCR mRNA expression analysis of several *P. berghei* genes (Figure 1C). Moreover, merosome-like structures with the characteristic labeling pattern of parasite DNA and the absence of host nucleus were repeatedly found in infected cultures (Figure 1B, d). Thus, *in vitro* infection of HC-04 cells with *P. berghei* ANKA GFP can serve as a model experimental system to study various aspects of malaria parasite interactions with the host cell at the exoerythrocytic stage of infection. We chose to utilize this model in order to dissect the MHC class I antigen presentation machinery in infected hepatocytes.

### Transcription of critical components of the MHC class I pathway is not altered in malaria-infected hepatocytes

**Figure 2:**
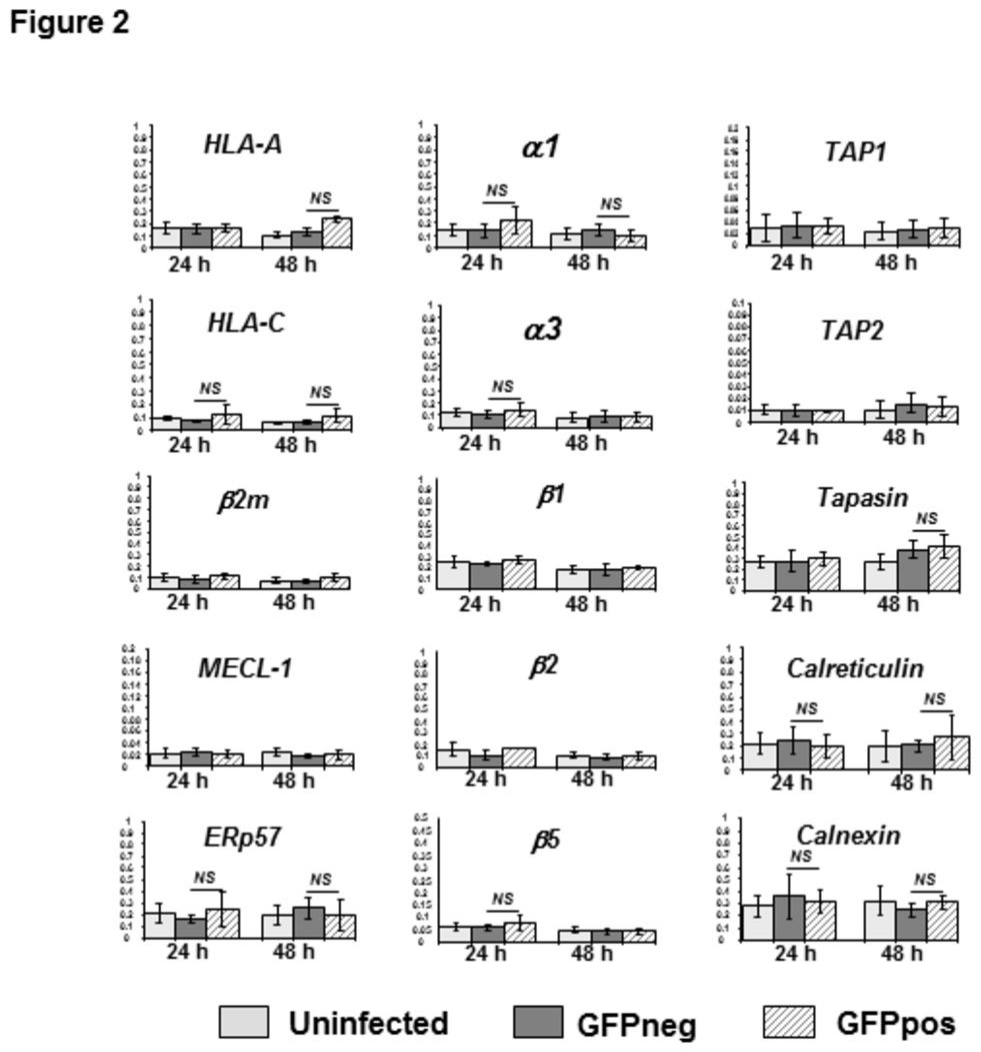
MHC class I pathway gene expression in hepatocytes infected with *P. berghei* ANKA GFP. GFP+ and GFP- HC-04 cells were isolated by FACS sorting at 24 and 48 hrs post-infection from the same parasite*-*infected cell cultures. Uninfected cells were also subjected to sorting prior to mRNA isolation. Real time PCR analysis of mRNA expression was done for the 15 genes indicated. Each assay was performed in triplicate. Data are shown as expression units relative to GAPDH expression levels arbitrarily taken as 1 and represent the mean ± SD from 4 to 9 independent infection experiments and cell sorting procedures. NS, not significant.

The MHC class I antigen presentation pathway represents a highly coordinated chain of events that includes proteolytic antigen processing, transport of antigenic peptides to the endoplasmic reticulum, MHC class I complex assembly and transport to the cell surface (reviewed in [23,24,43]). A malfunction at any single step of this pathway may result in downregulation of the final product, i.e. assembled MHC: peptide complexes, at the cell surface. We first examined whether or not transcription of genes critical for MHC class I processing and presentation is altered in the presence of rapidly developing malaria parasites. Using real-time RT-PCR analysis, levels of mRNA expression of seventeen such genes were assessed in HC-04 cells at 24 and 48 hours post-infection with *P. berghei* ANKA GFP as compared to that of GFP negative cells from the same sporozoite-exposed cultures as well as uninfected HC-04 cells (Figure 2 and data not shown). Expression of the following genes was compared in sets of samples derived from 4 to 7 independent infection experiments: α1 and α3, constitutive non-proteolytic proteasomal subunits; MHC class I heavy chains encoded by HLA-A and HLA-C loci; beta-2-microglubulin (β_2_m); β1, β2 and β5, proteolytic subunits of constitutive proteasomes; MECL-1, LMP2 and LMP7, proteolytic subunits of immune proteasomes, all reported to be involved in the generation of peptide epitopes; TAP1 and TAP2, transporters of peptides into the ER; tapasin, a component of the peptide loading complex; calnexin, calreticulin and ERp57, ER chaperones facilitating complex assembly and stability. Malaria parasite replication did not cause any significant changes in mRNA expression of the analyzed genes. LMP2 and LMP7 mRNAs were not detected in either uninfected or GFP negative and GFP positive cells from infected cultures (data not shown).

### Malaria infected hepatocytes retain expression of MHC class I complexes at the cell surface

**Figure 3:**
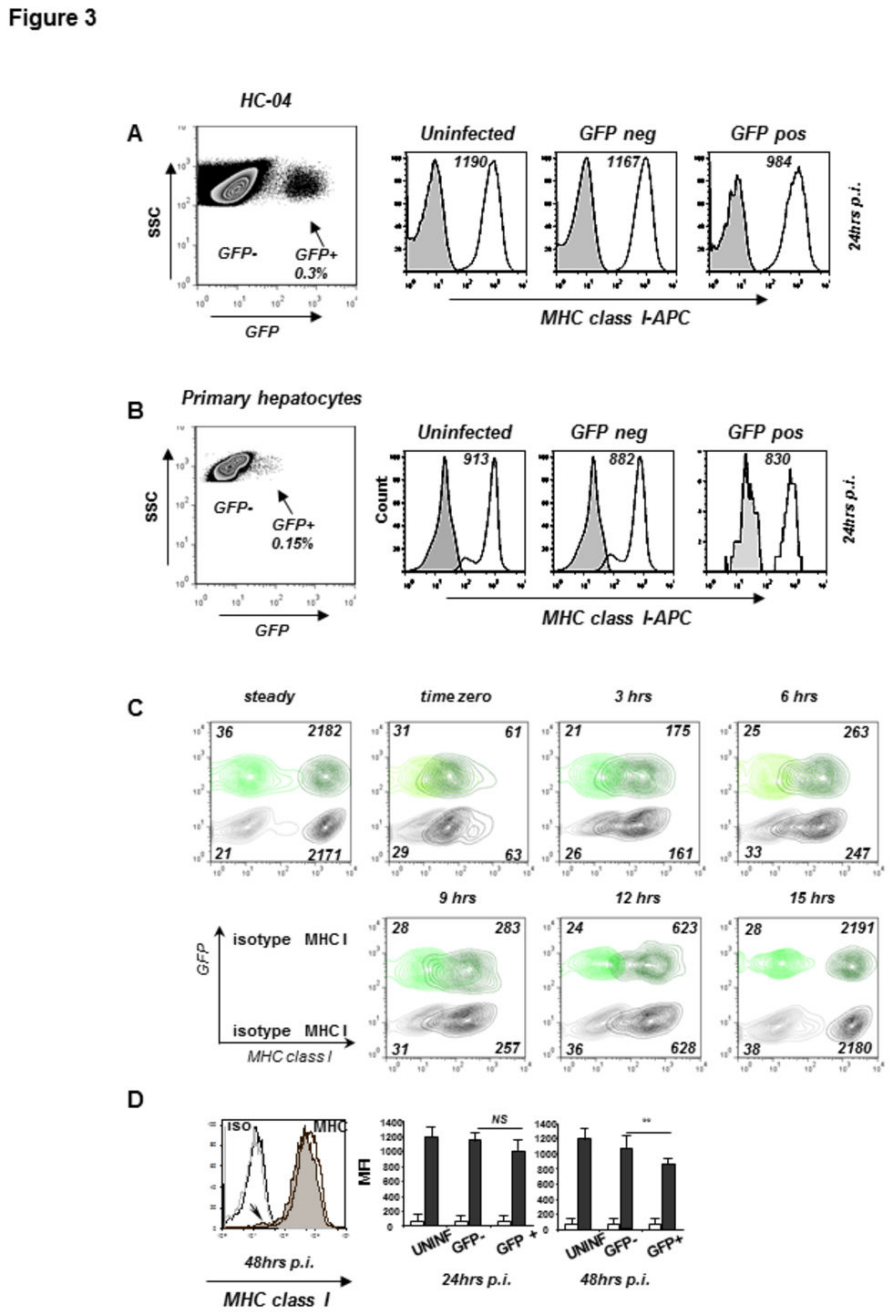
Surface MHC class I expression on infected hepatocytes at the early stages of parasite development. HC-04 cells (A, C, D) or primary freshly isolated human hepatocytes (B) were infected with *P. berghei* ANKA GFP and surface MHC class I was assessed by flow cytometry in uninfected or GFP+ and GFP- cell populations at 24 and 48 hrs post-infection. The dot plots (A and B) show the proportion of infected (GFP+) cells at 24 hrs post-infection. (A) One representative experiment performed at 24 hrs post-infection. Filled histograms - staining with the isotype control antibody; unfilled - staining with the MHC class I-specific antibody. The numbers are mean fluorescence intensities (MFIs) of MHC class I staining. (B) MHC class I expression on the surface of GFP+, GFP- and uninfected primary human hepatocytes. Filled histograms, staining with the isotype control antibody; solid lines, staining with the MHC class I-specific antibody. (C) Parasite replication does not affect *de* *novo* formation and transport of MHC class I complexes to the cell surface. HC-04 cells were subjected to treatment with a low pH buffer at 24 hrs post-infection to dissociate surface MHC class I molecules (time zero) and surface MHC class I expression was determined after the indicated periods of time post treatment. Overlay of contour plots for GFP+ and GFP- cells stained with the isotype control or MHC class I-specific antibody at each time point. Numbers indicate MFI in each population. One of three reproducible experiments. (D) MHC class I expression in GFP+ (filled histogram) and GFP- (unfilled histogram) populations 48 hrs post-infection with *P. berghei*. The arrow within the plot marks the small population of MHC class I negative cells. The bar-graphs show the means ± SD of MFI values obtained in 6 (for 48 hrs) and 9 (for 24 hrs) separate experiments. White and black bars represent staining with the isotype control or MHC class I-specific antibody, respectively. NS, not significant; p** = 0.03.

It is well established that activation of CD8+ CTLs is strongly influenced by the density of specific ligands at the surface of antigen presenting cells. Therefore, the availability of assembled MHC class I complexes define the efficacy of T cell activation. Taking advantage of GFP expression as a reliable marker of malaria parasite replication, we directly assessed expression MHC class I proteins at the surface of HC-04 cells (Figure 3A) exposed to *P. berghei* ANKA GFP sporozoites. Cells were analyzed by flow cytometry at 24 hrs post-infection following staining with antibodies specific to assembled MHC class I complexes. No significant differences in the levels of surface MHC class I were detected at 24 hrs post-infection in GFP+ or GFP- cell populations as compared to that of uninfected HC-04 cells. Similar results were obtained upon infection of primary human hepatocytes with *P. berghei* ANKA GFP (Figure 3B).

To investigate whether malaria parasite replication selectively affects only *de novo* formation of MHC class I complexes and thus does not have a significant impact on the total pool of MHC class I within the first 24 hrs post-infection, we analyzed MHC class I complex re-appearance at the surface of GFP- and GFP+ HC-04 cells from *P. berghei* ANKA GFP-infected cultures after a short incubation in a low pH buffer that results in dissociation of cell surface MHC class I molecules. As shown in Figure 3C, the kinetics of surface MHC class I reconstitution after low pH treatment in GFP+ cells did not differ from that in GFP- cells. This suggests that CTL peptide epitopes derived from the gene products *de novo* generated in malaria infected cells may be presented at the cell surface for recognition by the MHC class I restricted T cells.

The vast majority of infected HC-04 cells analyzed at 48 hrs post-infection exhibited only a relatively minor, 15-20 percent on average, decrease in the total level of surface MHC class I expression per cell (Figure 3D). However, already at this time point of parasite development, we consistently found a minor population of cells, varying in different experiments from 5 to 12 percent of all GFP+ cells, which were virtually MHC class I negative (Figure 3D, lower panel). Thus, our data demonstrate that development of *P. berghei* ANKA GFP in human hepatocytes does not significantly alter the density of MHC class I complexes at the surface of infected host cells. However, in a proportion of infected cells replication of the parasite results in nearly complete loss of MHC class I expression that is probably associated with the most terminal stages of sporozoite to merozoite transition.

### The circumsporozoite protein does not interfere with the steady-state or lymphokine-induced expression of MHC class I molecules

**Figure 4:**
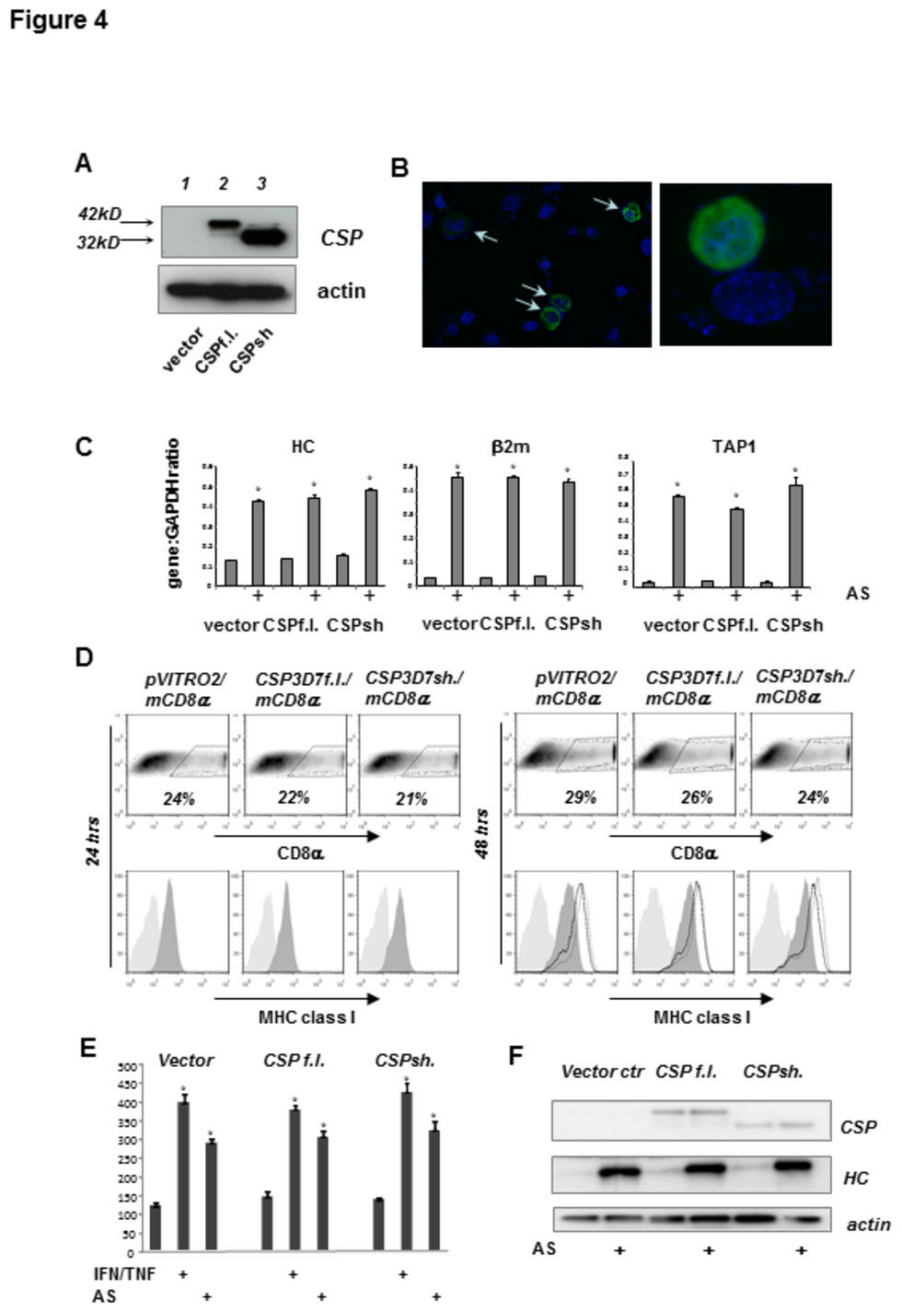
Circumsporozoite protein does not affect basal or inducible expression of MHC class I. (A) Expression of the full length (CSPf.l.) or mature “short” (CSPsh) form of the *P. falciparum* 3D7 CSP protein was detected by western blotting 24 hrs after transfection of HC-04 cells. (B) Cellular distribution of full length CSP 24 hrs post-transfection was visualized with a CSP-specific antibody (green) and immunofluorescence microscopy. Nuclei were stained with DAPI (blue). Arrows indicate transfected cells. (C) Real-time PCR analysis of MHC class I heavy chain, β2-microglobulin and TAP1 gene expression in cells transiently expressing of *P. falciparum* CSP. HC-04 cells transfected with the control vector plasmid or plasmid encoding either full length or short CSP were treated with AS (10% v/v) 4 hrs post transfection or left untreated. Transfected cells were isolated 24 hrs later using surface expression of mouse CD8α as a marker. Mean ± SD of the assay triplicates. All p* <0.0002 and indicated differences between control and AS-treated samples. (D) Percentages of cells transiently expressing CSP were identified by CD8α co-expression (upper panels) and MHC class I was assessed by flow cytometry (lower panel, light gray histograms - isotype control, dark gray histogram - MHC class I specific antibody). A proportion of cells exposed to either AS (10% v/v) or a mixture of recombinant TNFα and IFNγ at 24 hrs post transfection was further assessed for MHC class I expression at 48 hrs (light gray histograms - isotype control antibody, dark gray histogram - MHC class I in untreated cultures, solid line histogram – cultures treated with recombinant cytokines, dotted line histogram – cultures treated with AS). Data from one representative experiment. (E) The means ± SD of MFI for specific MHC class I staining obtained in 3 independent experiments. All p* <0.0001 and indicated differences between control and treated samples. (F) Expression of MHC class I heavy chain (HC) in total cell lysates of HC-04 cells transfected with CSP-expressing plasmids was assessed by western blot. Treatment with AS was done as described for D.

Upregulation of MHC expression by APCs in response to pro-inflammatory lymphokines plays a critical role in orchestrating efficient immune response against infections. Several pathogens are able to evade immune surveillance by interfering with lymphokine signaling through various molecular mechanisms (reviewed in [58]) The circumsporozoite protein (CSP), one of the most abundant proteins expressed by malaria sporozoites, has been shown to prevent TNFα-induced translocation of NF-κB into the nucleus of HepG2 or HeLa cells thereby inhibiting transcription of NF-κB responsive genes [59]. These results prompted us to investigate whether CSP expression alone could be sufficient to inhibit the inducible expression of genes controlling antigen processing and presentation. To closely examine the effect of CSP on MHC class I expression, we ectopically expressed the two major forms of CSP from *P. falciparum* 3D7: the full length protein (CSPf.l.) and its truncated “mature” form (CSPsh), produced by the proteolytic cleavage during the sporozoite’s interaction with the plasma membrane of host hepatocyte [60] (Figure 4A, B). CSP expression was driven from a bicistronic plasmid vector also encoding mouse CD8α (mCD8α) whose presence on the cell surface as a unique marker for transfection allowed detection and/or sorting of CSP+ cells. Using this approach HC-04 cells expressing mCD8α alone or co-expressing it either with CSPf.l or CSPsh were isolated 24 hrs after transfection or directly detected using immunostaining and flow cytometry. In the course of natural immune response, hepatocytes are likely to be exposed to a plethora of different lymphokines produced by activated T-cells. Therefore, we analyzed the modulation of the MHC class I machinery in either mCD8α vector control, CSPf.l. or CSPsh plasmid transfected HC-04 cells not only after treatment with a combination of recombinant TNFα and IFNγ but also following incubation with supernatant collected from human activated CTLs. Neither CSPf.l nor CSPsh expression affected the steady-state or cytokine-induced mRNA levels of MHC class I heavy chain, β_2_m or TAP1 mRNA assessed 24 hrs after transient transfection of HC-04 cells (Figure 4C). In line with this observation, expression of CSPf.l. or CSPsh did not modulate MHC class I expression at the surface of HC-04 cells cultured in medium alone or in the presence of either recombinant human IFNγ and TNFα or supernatant of activated human CTLs (Figure 4D and 4E). Likewise, expression levels of MHC class I heavy chains assessed by immunoblotting of total cell lysates of HC-04 cells transfected with the CSPf.l or CSPsh encoding plasmid were comparable to that observed in lysates of cells transfected with the control vector (Figure 4F). Collectively, these results indicate that the expression of CSP does not interfere with the antigen presenting machinery in human hepatocytes.

### MHC class I upregulation in response to pro-inflammatory lymphokines is unaltered in malaria infected hepatocytes

**Figure 5:**
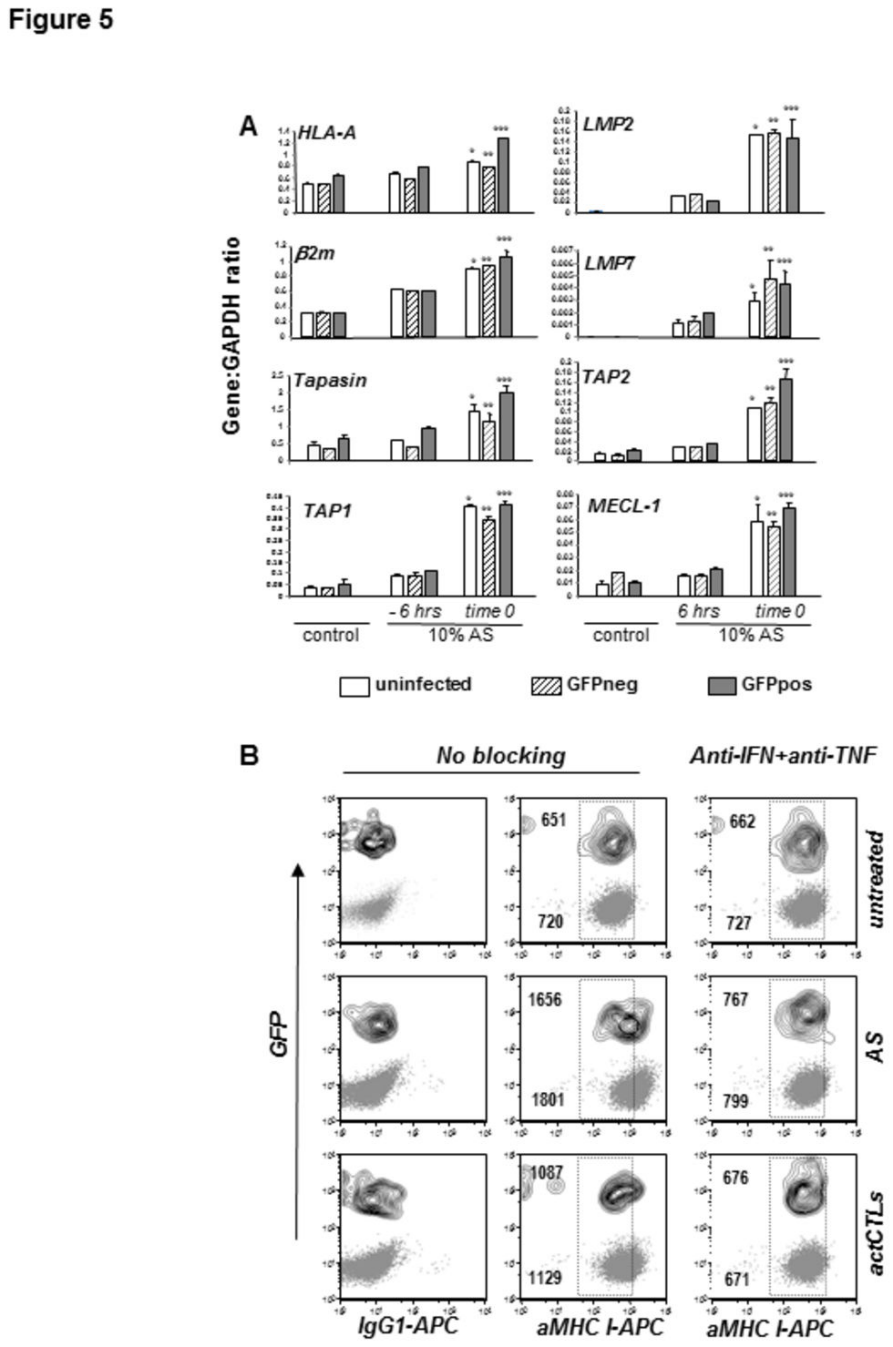
Effect of activated CTLs on the MHC class I pathway in infected hepatocytes. (A) Expression of the genes involved in the MHC class I pathway was monitored by real-time PCR in uninfected, GFP+ and GFP- HC-04 cells in response to treatment with AS as described in the legend of Figure 2. HC-04 cells were either pre-incubated with AS for 6 hrs prior to infection with *P. berghei* ANKA GFP, or AS was introduced to the infected cultures 2 hrs post-infection. Data are shown as expression units relative to *GAPDH* housekeeping gene expression levels arbitrarily taken as 1 and represent means ± SD from 3 individual infection and cell sorting procedures. All *p* values are <0.0005 and indicate differences between control and treated uninfected (*), GFP negative (**) and GFP positive (***) cells. (B) Human CTLs were pre-activated on third party targets as described in Materials and Methods. HC-04 cultures infected with *P. berghei* ANKA GFP were either cultured in complete medium (untreated), exposed to AS or co-cultured with pre-activated CTLs (1:100 CTL to hepatocyte ratio). Additionally, each of the three cultures was incubated with IFNγ and TNFα blocking reagents (anti-TNF/anti-IFN) or left untreated (no blocking). Surface MHC class I staining was done on infected cultures at 48 hrs post-infection. Dot plots (GFP-) and contour plots (GFP+) represent MHC class staining with an APC-labeled MHC class I specific antibody or relevant isotype control antibody on infected and uninfected hepatocytes, respectively. One representative of 3 experiments is shown. The numbers within plots show MFI of MHC class I specific staining for the indicated culturing condition and cell subpopulation.

As we failed to observe any significant effects of ectopic CSP expression on lymphokine-induced upregulation of MHC class I in HC-04 cells, we set out to investigate whether or not such effects are caused by malaria parasites in the course of their replication in the host cell (Figure 5A). We analyzed mRNA expression of the heavy and light chains of the MHC class I complex, peptide transport and loading complex consisting of TAP1, 2 and tapasin, as well as three cytokine-inducible subunits of immuneproteasome, LMP2, LMP7 and MECL-1 in GFP+ (infected) hepatocytes following treatment with the soluble factors released by activated CTLs. Addition of activated CTL supernatant (AS) at “time zero” of infection, and, to a lesser extent, pre-treatment of hepatocytes for 6 hrs prior to infection, resulted in upregulation of mRNA levels of all tested genes in uninfected, GFP- or GFP+ cells. Hence, hepatocytes supporting development of the parasite preserve the capacity to respond to soluble factors produced by activated CTLs upregulating transcription of genes critically involved in MHC class I antigen presentation.

To identify whether transcriptional upregulation of the above mentioned genes results in changes of surface MHC class I density on infected cells, we exposed infected cultures to either pre-activated EBV-specific CTLs, which do not recognize HC-04 cells as targets, or to AS at 2 hrs post-infection. We found that both types of treatment triggered comparable MHC class I induction in GFP+ and GFP- hepatocytes that was abolished by a combination of IFNγ- and TNFα-blocking agents (Figure 5B). Collectively, these results demonstrate that pro-inflammatory cytokine-mediated regulation of the MHC class I pathway is not impaired in malaria-infected hepatocytes at the early stages of parasite development.

**Figure 6:**
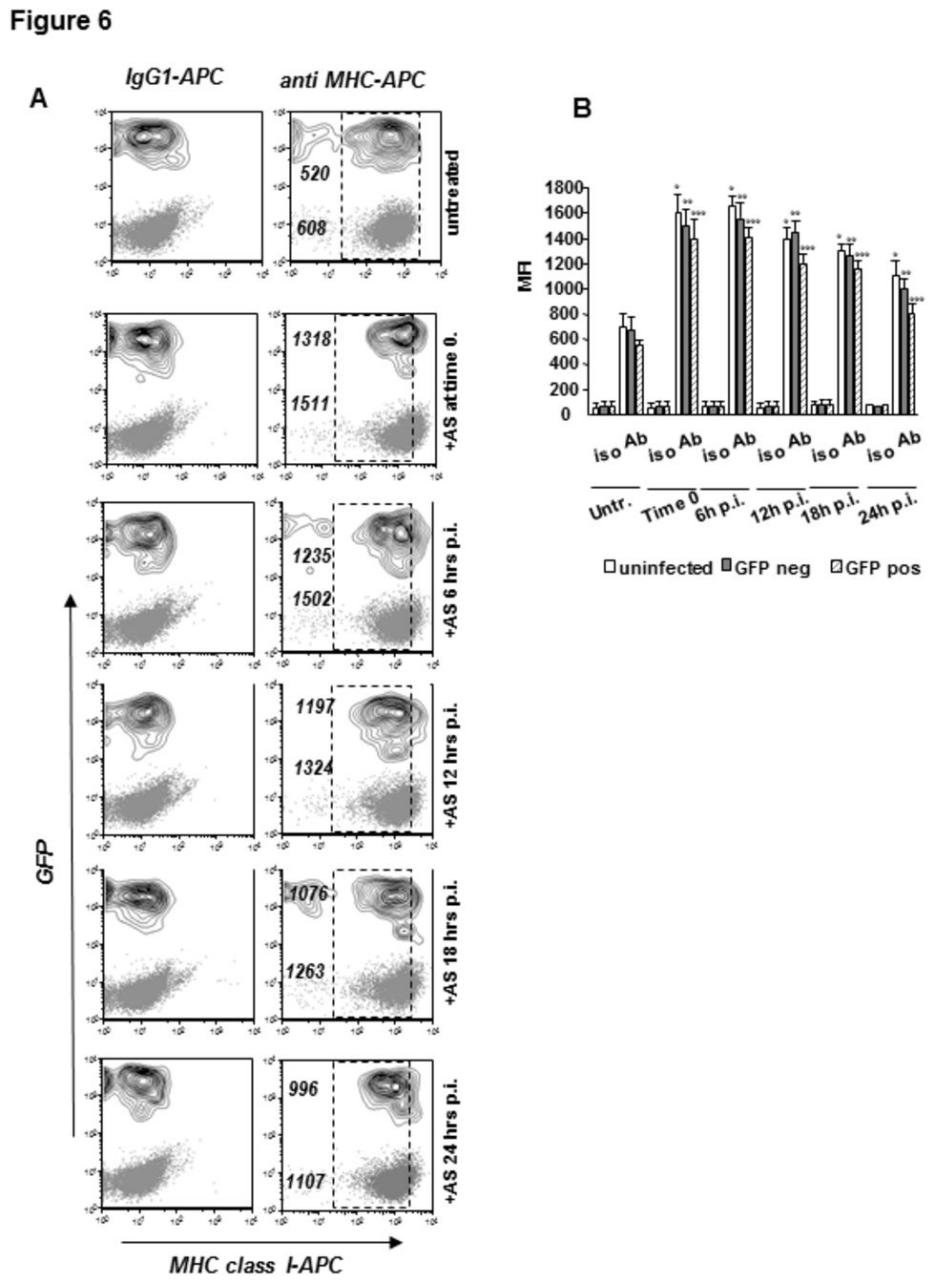
Kinetic analysis of AS-mediated effects on MHC class I expression in infected hepatocytes. HC-04 cultures infected with *P. berghei* ANKA GFP were exposed to activated supernatant (AS, 10% v/v) at the indicated time points post-infection and surface MHC class I staining was performed at 48 hrs post-infection. (A) Dot plots (GFP-) and contour plots (GFP+) represent MHC class staining with an APC-labeled MHC class I specific antibody or relevant isotype control antibody on infected and uninfected hepatocytes, respectively. One representative of 3 experiments is shown. The numbers indicate MFI values of MHC class I specific staining for respective conditions and populations. The dotted line boxes mark the observed area of events distribution in untreated cultures. (B) Summary of 3 experiments performed as described in A. The means ± SD of MFI values for surface MHC class I staining are shown. All *p* values are ≤0.01 and indicate differences between control and treated uninfected(*), GFP negative (**) and GFP positive (***) cells.

We next analyzed whether malaria parasite replication can counteract induction of MHC class I expression at later stages of infection (Figure 6). We found that GFP+ and GFP- HC-04 cells from the same infected cultures upregulated MHC class I at the cells surface to comparable levels in response to AS administered as late as 24 hrs post-infection, when exoerythrocytic forms of *P. berghei* ANKA GFP are rather advanced in their development and infected cells already contain large PVs of high integrity as shown in Figure 1. Similar to our previous findings, surface MHC class I density before exposure to pro-inflammatory lymphokines was slightly lower on GFP+ as compared to GFP- cells. However, the vast majority of infected cells expressed and further upregulated MHC class I in response to AS as detected at 48 hrs post-infection. Thus, human hepatocytes, which support late stages of malaria parasite development, remain sensitive to pro-inflammatory cytokine signaling and can be targeted to modulate the MHC class I antigen processing pathway throughout most of the timespan of exoerythrocytic form maturation.

### MHC class I complexes are detected on the surface of human hepatocytes supporting development of *P. falciparum*

**Figure 7:**
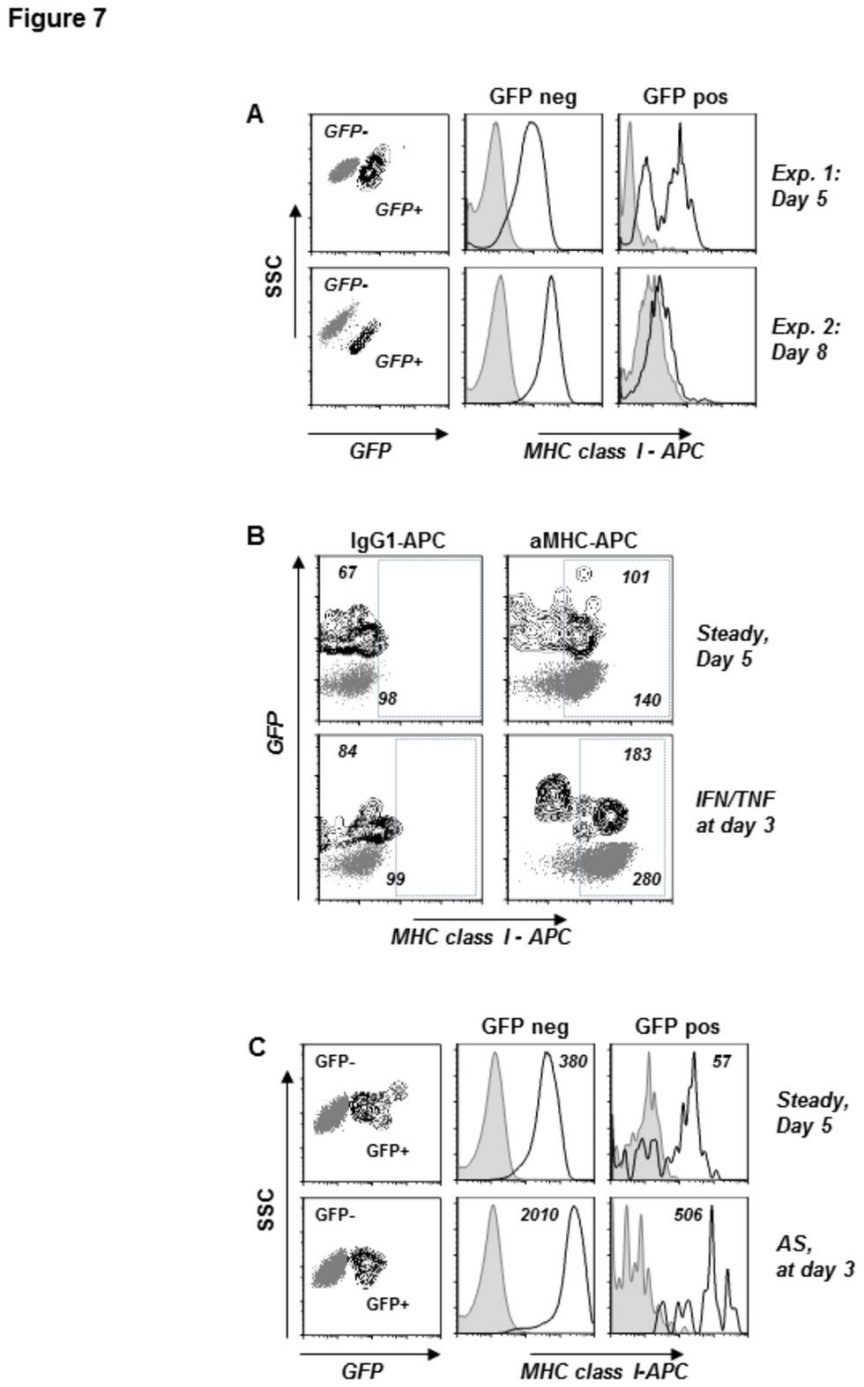
Effect of *P. falciparum* 3D7 GFP infection on surface MHC class I expression. Primary human hepatocytes were infected with *P. falciparum* 3D7 GFP sporozoites and surface MHC class I expression was assessed in GFP+ and GFP- cell populations at days 5 and 8 post-infection using immunostaining followed by flow cytometry (for details see Materials and Methods). (A) Dot plots (gray) show side scatter characteristics of GFP- cells (uninfected), while the contour plots (black) show GFP+ (infected) populations at day 5 and day 8 post-infection. Filled histograms represent staining with an isotype control antibody; solid lines, staining with a MHC class I-specific antibody in both populations at day 5 and day 8. Data are representative of two independent experiments. (B) Modulation of MHC class I in *P. falciparum* 3D7 GFP infected primary hepatocytes on day 5 post-infection by recombinant IFNγ and TNFα supplied at day 3 post-infection. Dot plots (GFP-) represent populations of uninfected cells, while contour plots show infected (GFP+) cells. Numbers are MFI of specific fluorescence obtained following staining with an APC-conjugated isotype control or MHC class I-specific antibody. (C) Expression of MHC class I in *P. falciparum* 3D7 GFP infected primary hepatocytes on day 5 post-infection in response to AS (10% v/v) supplied on day 3. Filled histograms show staining obtained with an isotype control antibody, solid lines - staining obtained with an MHC class I specific antibody. Numbers represent MFI values of cell populations stained for MHC class I expression.

In the experiments described above, we took advantage of the ability of human HC-04 hepatocytes to support the development of *P. berghei* ANKA GFP liver forms as well as the relative ease of obtaining large amounts of purified *P. berghei* sporozoites (20-30 x 10^6^ in each isolation procedure) followed by detection of infected hepatocytes based on GFP expression. To verify our key observations with a biologically relevant combination of human hepatocytes with a human malaria parasite, we turned to a recently developed GFP-expressing human parasite strain, *P. falciparum* 3D7-GFP [54]. As the durations of the *P. falciparum* and *P. berghei* exoerythrocytic developmental cycles differ significantly (6-8 and about 2 days for the human versus rodent parasite, respectively) both *in vitro* and *in vivo*, we monitored surface MHC class I expression on HC-04 cells, as well as primary human hepatocytes infected with *P. falciparum* 3D7 GFP at day 5 and day 8 post-infection. Our multiple attempts to detect GFP signal in HC-04 cultures through days 1 to 3 post-infection failed, whereas the first GFP-positive cells with dim fluorescence were detected only in some of our experiments on day 4 post-infection (data not shown). Similar to the data obtained with *P. berghei* at 48 hrs post-infection (Figure 5), the vast majority of *P. falciparum* infected primary human hepatocytes displayed MHC class I at the cell surface at day 5, whereas at day 8 post-infection, virtually all detectable GFP+ cells lost their surface MHC class I (Figure 7A, B). Recombinant IFNγ and TNFα (Figure 7B) as well as activated T-cell supernatant (Figure 7C) administered on day 3 enhanced MHC class I expression at the surface of hepatocytes infected with *P. falciparum* 3D7 GFP as detected at day 5 post-infection, indicative of unimpaired pro-inflammatory lymphokine response in *P. falciparum* infected hepatocytes.

### Malaria infected hepatocytes trigger activation of human cytotoxic T cells

**Figure 8:**
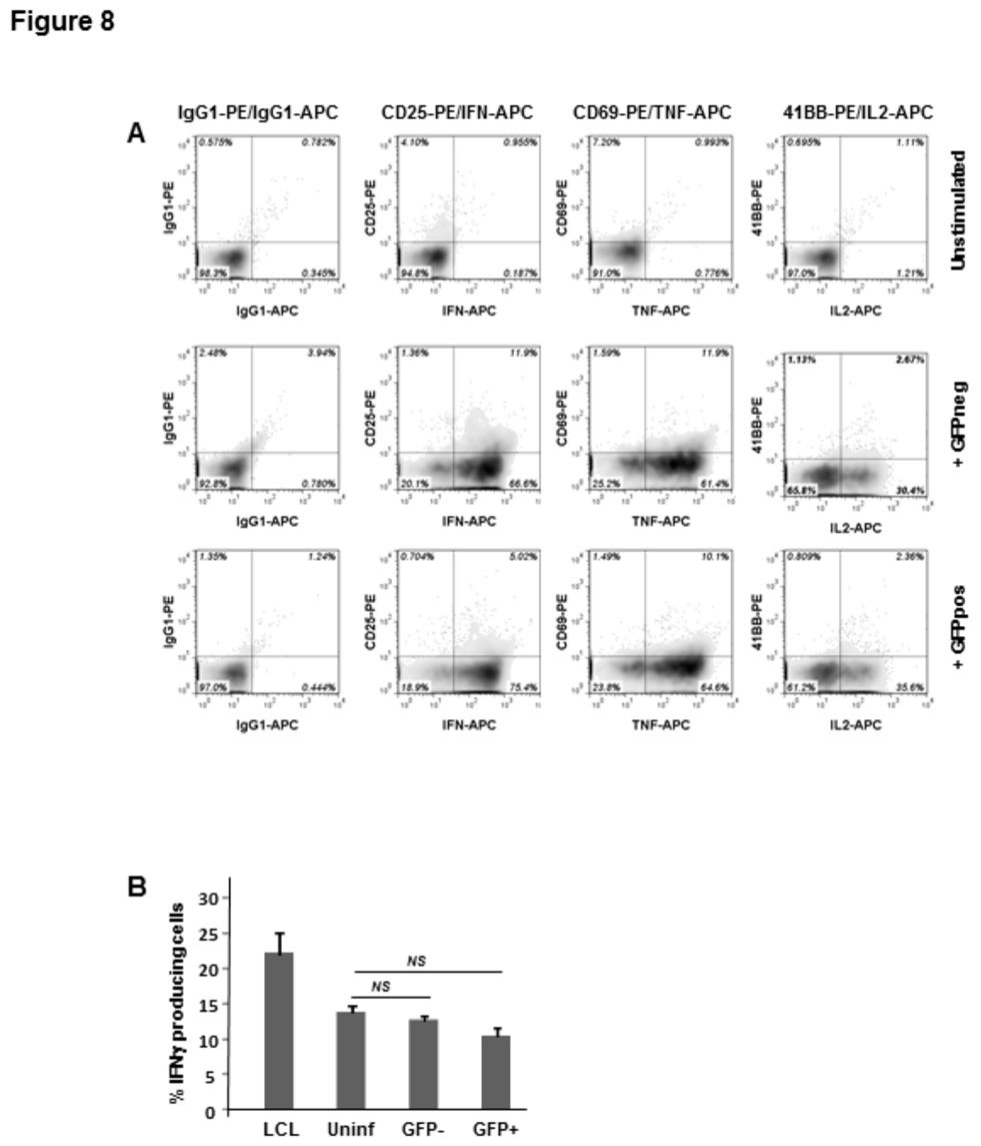
Activation of CTLs exposed to human hepatocytes infected with *P. berghei* ANKA GFP. (A) Activation of GLC peptide specific CTLs was done using uninfected, GFP- and GFP+ HC-04 cells as stimulators as described in Materials and Methods. One representative experiment of 3 performed is shown. Numbers indicate percentages of positive cells for each activation marker indicated in the figure. (B) Cytokine release from HLA-A2 specific allogeneic T cells activated on infected and uninfected HC-04 cells is described in Materials and Methods. Summary of 3 independent experiments. HLA-A2 positive LCL was used as a control of T cell activation. NS, not significant.

We next asked whether unaltered MHC class I expression on *Plasmodium* -infected cells confers them with the capacity to specifically activate human CTLs. Uninfected and infected cells do not, presumably, share an intracellular antigen capable of inducing natural CD8+ T cell responses. Therefore, to be able to compare the ability of these targets to serve as inducers of T cell activation, we took advantage of the potency of peptide specific HLA-restricted CTLs to recognize HLA-matched targets pre-pulsed with the synthetic peptide epitope. HLA A2-restricted peptide-specific (Figure 8A), as well as allogeneic HLA A2-specific (Figure 8B) CD8+ T cells were assessed for multiple markers indicative of T cell activation upon co-culture with GFP+, GFP- or uninfected HLA A2+ HC-04 cells. No significant difference in the production of cytokines was observed upon stimulation of two different T cell clones with *P. berghei* infected or uninfected hepatocytes (Figure 8) Thus, human hepatocytes supporting development of malaria parasites show no alterations in their capacity to induce activation of MHC class I restricted effector T-cells.

### MHC class I negative population arising in the course of malaria infection is composed of merosome-like structures

**Figure 9:**
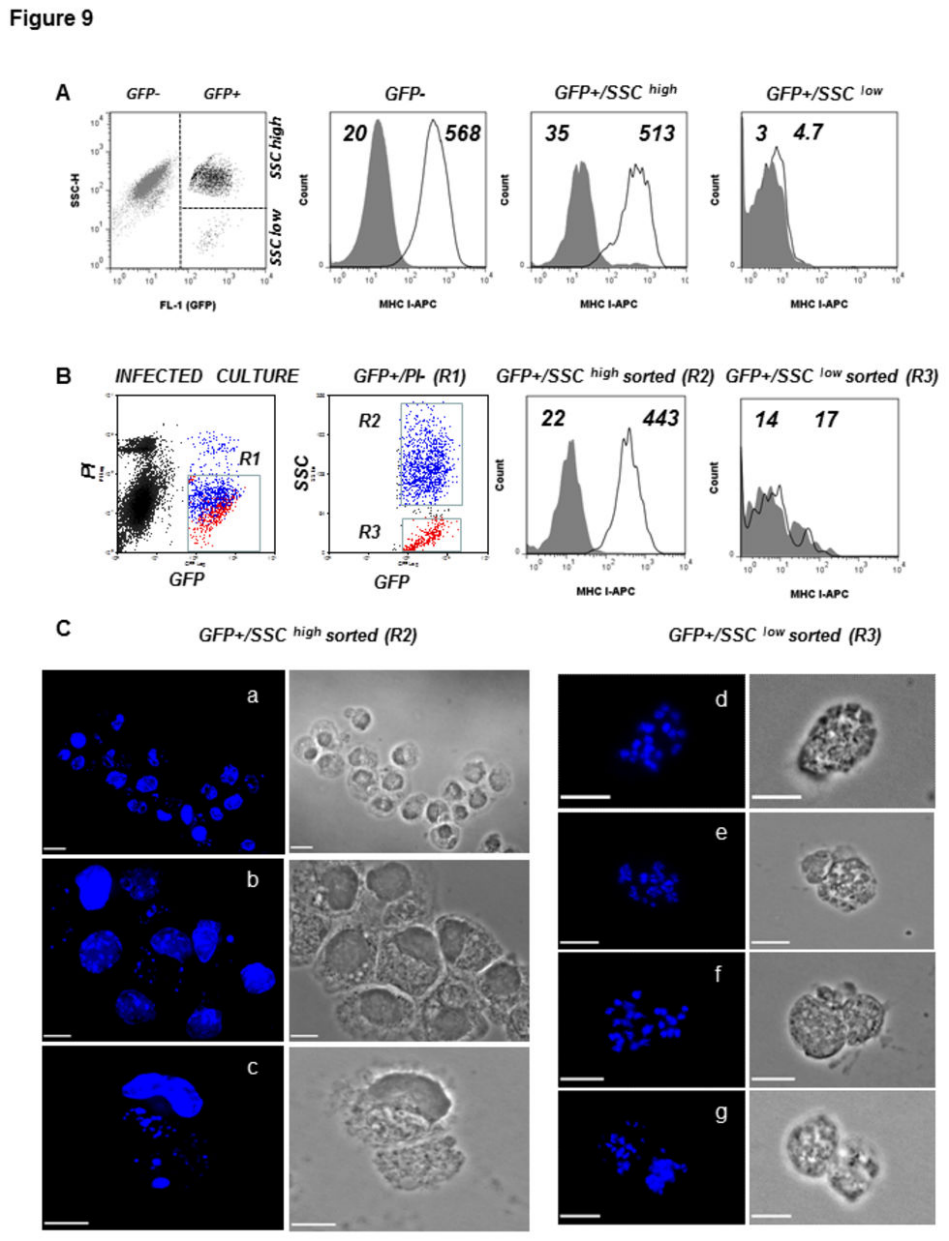
Identification, isolation and MHC class I expression on merosomes. MHC class I expression in GFP+/SSC^low^ population was assessed by flow cytometry in unsorted (A) and sorted (B) *P. berghei* ANKA GFP infected HC-04 cultures. (A) The dot plot graph demonstrates identification of GFP-, GFP+/SSC^high^ and GFP+/SSC^low^ populations. The histograms demonstrate MHC class I expression (solid line) and staining with an isotype control antibody (filled histograms). The numbers indicate values of relevant MFI. (B) GFP+/PI- HC-04 cells (R1) were separated into GFP+/SSC^high^ (R2) and GFP+/SSC^low^ (R3) by cell sorting and MHC class I expression was monitored in each population separately. In parallel, cells were observed by immunofluorescence. (C) Cytospin preparations of sorted cells or subcellular structured from gates R2 (a,b,c) and R3 (d,e,f,g) as shown on panel B were fixed and mounted with DAPI-containing mounting media. Presence of host and parasite-derived DNA was monitored using a Nikon90i microscope at 40X (a) and 100X (b-g). Scale bars indicate 20 µm (a) and 10 µm (b-g).

Albeit at different kinetics, the appearance of MHC class I negative GFP+ cell populations was observed in HC-04 cultures following infection with either *P. berghei* (Figure 3D) or *P. falciparum* (Figure 7). We set out to analyze in more detail the characteristics of this subpopulation as potential immune escape variants. We noted that at 36 to- 48 hrs post-infection *P. berghei* development in human hepatocytes gave rise to the GFP+ PI- cell pool that was divided into side scatter (SSC)^high^ and SSC^low^ populations on flow cytometry plots, the latter representing 3-10% of infected cells (Figure 9A, B and data not shown). We measured surface MHC class I expression of this population in total infected cultures (Figure 9A) as well as following cell sorting (Figure 9B) and found no detectable surface MHC class I in the GFP+/SSC^low^ compartment. We next observed that in contrast to GFP+/SSC^high^ cells containing both the host nucleus and developing parasite (Figure 9C panels a, b, c), the GFP+/SSC^low^ pool was represented by remarkably different structures devoid of the host nucleus, exhibiting a dotted pattern of DNA staining (Figure 9C panels d, e, f, g) and MSP-1 expression (data not shown), all characteristic features of merosomes. To better understand the dynamics of MHC class I in hepatocytes infected with *P. berghei* parasite, we extended the time kinetic of MHC class I analysis to 4 days post infection and observed that similar to the pattern seen with *P. falciparum* (Figure 7), nearly the entire GFP+ cell population lost surface MHC class I expression at 96 hrs post infection (Figure S2). The latter was accompanied by a drop in cellular granularity (SSC characteristics) and a decrease in intensity of GFP signal, probably reflecting the variety of transient phenotypes of host cell/parasite-derived populations.

Thus, though it had been recently demonstrated that merosome membrane derives from the host cell membrane [61], our data strongly suggest that merosomes formed at the terminal stages of parasite development in the liver cannot be targeted by T cell mediated immunity through MHC class I.

## Discussion

In this study, we report the first characterization of the MHC class I processing and presentation pathway in malaria infected hepatocyes. Although the issue is of the utmost significance for our understanding of T-cell mediated control of malaria infection and vaccine development, it has not been systematically addressed due to the lack of appropriate experimental models. Here we capitalized on the availability of transgenic GFP-expressing malaria parasites and their capacity to undergo the full program of sporozoite to merozoite transition in HC-04 cells within the time frame comparable to that observed *in vivo* during the liver stage of malaria infection. We showed that HC-04 cells are permissive not only for *P. falciparum* and *P. vivax*, as has been reported previously [53], but support *P. berghei* replication as well (Figure 1). This combination of a rapidly replicating malaria parasite with permissive immortalized hepatocytes of human origin created a novel, efficient experimental system allowing phenotypic and biochemical characterization of MHC class I expression in a sizable population of malaria infected hepatocytes in a controlled and reproducible manner.

Following infection with *P. berghei* sporozoites, mRNA expression levels of all major components of the MHC class I processing and presentation machinery were not significantly affected by parasite replication as assessed at 24 or 48 hours post-infection (Figure 2). Furthermore, the levels of MHC class I expression were decreased only by 10-20 percent at the surface of infected host cells as compared to either uninfected or untreated controls (Figure 3). Notably, a well-defined parasitophorous vacuole encompassing multiple developing parasites was observed already at 24 hours post-infection in all infected nucleated cells isolated by FACS sorting based on expression of parasite-encoded GFP (Figure 1). Altogether, these results unequivocally showed that the process of *P. berghei* replication from its initiation to complete formation of the parasitophorous vacuole has no major effects on the steady state levels of MHC class I expression in HC-04 cells.

Based on the reported inhibitory effect of CSP on TNFα-induced NF-κB translocation into the nucleus of HepG2 or HeLa cells [59], it has been suggested that malaria infection may interfere with inducible expression of MHC class I in hepatocytes. We set out to revisit this idea using the experimental system based on parasite replication-permissive HC-04 cells. Results presented in Figure 4 clearly show that ectopic expression of either the full length or short form of CSP does not affect the steady state expression levels of MHC class I in HC-04 cells. Likewise, CSP did not interfere with upregulation of MHC class I expression induced by exposure of HC-04 cells to recombinant IFNγ and TNFα or supernatant of activated antigen specific CD8+ T-cells (Figure 5). The latter was used as a complex mixture of pro-inflammatory lymphokines to which hepatocytes might be exposed in the course of natural or vaccination-induced anti-malaria immune responses. Next, we investigated whether interference with lymphokine-induced upregulation of MHC class I can take place in the course of parasite replication in HC-04 cells. Exposure of infected cultures to supernatants of activated T-cells either before, concomitantly, or at several time points after infection with malaria sporozoites resulted in comparable up-regulation of MHC class I expression at the surface of infected (GFP+) or non-infected (GFP-) HC-04 cells (Figure 6). Therefore, our experiments produced no evidence for the capacity of the developing malaria parasite to suppress lymphokine inducible upregulation of MHC class I molecules either at the early or late stages of parasite development in HC-04 cells, as long as they retain the typical morphology of infected nucleated cells containing a large parasitophorous vacuole.

Collectively, our data do not support previously found existence of transcriptional and translational inhibition in mammalian cells infected with malaria parasites [59,62]. Several differences in experimental approaches may account for these discrepancies. First, data published by Singh et al. [59] were obtained using a different cellular background, human cervical epithelial cancer cell line HeLa, whereas our experiments were conducted in hepatocytes, a biologically relevant target of the liver stages. Second, Singh et al. induced ectopic CSP expression using prolonged treatment with doxycycline, an inducer of defective ribosomal products and ER-stress. Moreover, the authors did not discriminate between the cells expressing and not expessing CSP from the same doxycycline-treated culture. In contrast, we used the transient expression of CSP in the absence of additional drug stimuli and discriminated between CSP-transfected and non-transfected cells from the same culture, the latter was used as the most relevant and accurate negative control. Third, neither of the studies mentioned above performed comparison of two cell populations, infected and non-infected hepatocytes, separated following exposure to sporozoites. Finally, our current study was focused only on the major genes involved into MHC class I antigen processing and presentation and, while we did not find any defects in the expression of these genes in CSP-expressing mammalian cells, we cannot formally exclude that expression of other genes might have been affected by CSP. Nevertheless, any such changes did not result in alterations of the MHC class I pathway in human hepatocytes as demonstrated by our data.

To verify our key findings with a biologically relevant combination of human hepatocytes with a human malaria parasite, we monitored surface MHC class I expression on hepatocytes infected with the *P. falciparum* 3D7 GFP strain at days 5 and 8 post-infection. Consistent with the data obtained through the analysis of *P. berghei* infected HC-04 cells, primary human hepatocytes retained MHC class I surface expression at day 5 post-infection with *P. falciparum*. Moreover, infected and uninfected primary hepatocytes, exposed to pro-inflammatory lymphokines at day 3 post infection upregulated MHC class I molecules to a comparable extent (Figure 7). Thus, data generated through the analysis of either *P. berghei*-infected HC-04 cells or *P. falciparum*-infected primary human hepatocytes unequivocally showed that malaria parasite development from the entry of *Plasmodium* sporozoites to the formation of morphologically defined parasitophorous vacuole is not associated with significant downregulation of MHC class I molecules at the cell surface of infected cells. The notion that a 10 to 20% decrease of MHC class I expression observed in *P. berghei* infected HC-04 cells at 48 hours post-infection may have no important functional consequences was further supported by our demonstration that such cells exhibited virtually unaltered capacity to stimulate activation of peptide-specific or allogeneic HLA-A2-specific CD8+ T-lymphocytes (Figure 8).

Interestingly, infected cells exhibiting drastically reduced surface expression of MHC class I were detected at day 8 post-infection in *P. falciparum* 3D7 GFP infected cultures of primary human hepatocytes. Unfortunately, the detailed biochemical analysis of the MHC class I pathway or the characterization of T-cell stimulatory capacity could not be performed for these cells due to their low frequencies. It is conceivable that the “MHC class I low” subpopulation of hepatocytes is composed of infected cells undergoing the late phases of exoerythrocytic parasite development. A similar subpopulation of cells is likely to escape detection in *P. berghei* infected HC-04 cultures due to the faster rate of development (Figure 9 and Figure S2). In agreement with this notion, we identified and isolated subcellular structures with the typical morphology of merosomes from *P. berghei* infected HC-04 cell cultures that were devoid of detectable surface MHC class I expression monitored by sensitive flow cytometry based methods. Recently, using live cell imaging and fluorescence microscopy it was elegantly demonstrated that though the merosome membrane derives from the host cell membrane, the host proteins are lost following merozoite liberation from the parasitophorous vacuole [61]. Although previous data on MHC class I expression measured on fixed cells should be considered with caution, as common fixation procedures largely diminish antibody-based detection of surface class I complexes, our data based on quantitative specific measurement of assembled, peptide cargo-containing surface MHC class I complexes using the W6/32 antibody and performed simultaneously on different subpopulations of cells co-existing in malaria infected human cell cultures are in agreement with the findings of Graewe et al. [61]. Further analysis of “merosome-like SSC^low^ subpopulation” detected by flow cytometry in *Plasmodium* -infected cultures is needed; this, however, represents a technical challenge due to a small number and high vulnerability of these cells.

In summary, our results indicate that malaria infected hepatocytes, which represent the only reservoir of parasite replication during the liver stage of infection, retain unaltered steady state or inducible expression of surface MHC class I molecules as well as T-cell stimulatory capacity during most of the process of sporozoite to merozoites transition. This is consistent with the capacity of malaria specific CD8+ T-cells to control parasite development in vaccinated humans or animals. In the current study, in order to characterize the T-cell stimulatory capacity of malaria infected hepatocytes we utilized either human T cells recognizing peptide epitope provided exogenously or allogeneic HLA A2 specific CTLs capable of recognizing HLA-A2 structures regardless of specific peptide cargos (Figure 8). This approach allows monitoring of T-cells activated by the same ligands on both uninfected and infected hepatocytes. It cannot be formally excluded that the parasites have evolutionarily developed molecular mechanisms specifically targeting the processing and presentation of MHC class I complexes containing parasite-derived peptides. However, in agreement with the previously published evidence on recognition of CSP in a mouse model [11], we find such a scenario highly unlikely considering the lack of any evidence for biochemical alterations of the pathway in the course of malaria replication.

Emergence of MHC class I negative subpopulation at the late stages of parasite development has several important implications for the design of anti-malarial vaccines relying on specific peptide-epitope recognition by CD8+ T cells. *First*, it indicates that the efficacy of CD8+ CTL-mediated control over parasite replication in the liver may require both timely recruitment of and effector function acquisition by antigen specific T-cells migrating into the organ or the presence of pre-existing specific T cell infiltrates in the liver of the host. This assumption is in agreement with the well-documented protective effect of vaccination-induced MHC class I-restricted T cell responses that is contrasted by the apparent failure of T cells to control natural malaria infection. *Second*, our observations suggest that the repertoire of antigenic targets, which can be recognized by malaria specific CTLs in the liver, might be strongly skewed in favor of antigens expressed at rather early stages of exoerythrocytic development. Such antigens have a chance to be processed and presented for T cell recognition before the appearance of parasite-bearing structures with low or undetectable levels of surface MHC class I expression.

It is conceivable that malaria-infected hepatocytes are able to interfere with the activity of T-cell effector functions through mechanisms unrelated to changes in MHC class I expression. This notion has been indirectly supported by *in vivo* single cell imaging of CTL interactions with infected hepatocytes that led the authors to suggest that enhanced cytotoxic activity of parasite-specific CTLs is associated with their increased capacity to eliminate malaria infected targets [63]. Therefore, interactions of malaria infected hepatocytes with the cytotoxic and apoptosis inducing machineries of CTLs remain to be characterized in a quantitative manner. Research in this area should further define specific functional requirements for highly protective malaria specific T-cells.

## Supporting Information

Figure S1 **Amino acid sequences generated by the plasmids encoding CSP “full length” and CSP “short” of *P. falciparum3D7* strain.** CSP serves as a substrate for cysteine protease during parasite interaction with the host cell plasma membrane thus generating “short CSP” [60]. Methionine (M), serving as initiation of translation is underlined in each sequence.
(TIFF)

Figure S2 **Kinetic of surface MHC class I expression in infected hepatocytes.** Expression of surface MHC class I was measured in GFP positive population obtained from HC-04 cells infected with *P. berghei* ANKA GFP and sorted at 4 hrs post infection. Sorted cells were propagated in complete media at 37°C and MHC class I was measured at 48, 72 and 96 hrs post infection as described in Materials and Methods. Upper panels show SSC and GFP characteristics of sorted cells at each time point, arrows indicate SSC^high^ and SSC^low^ population at 48 hrs post infection. Note, that both SSC and GFP intensity values decreased over time of observation. Lower panels demonstrate surface MHC class I staining, filled histograms - isotype control antibody staining, unfilled histograms (marked by arrows) - surface MHC class I staining.
(TIF)

Table S1 **Genes verified by quantitative real time PCR.** The critical genes involved into the MHC class I pathway in HC-04 hepatocytes were verified by q-RT-PCR using the listed TaqMan® gene expression assays from (Applied Biosystems, CA, USA).
(TIFF)
